# High-risk HPV genotypes in women with abnormal cytology: a 12-year retrospective study

**DOI:** 10.1186/s13027-025-00664-0

**Published:** 2025-05-26

**Authors:** Masoumeh Aslanimehr, Shabnam Nemati, Hamid Sadeghi, Fatemeh Samiee-Rad, Sahand Ghafari, Taghi Naserpour-Farivar

**Affiliations:** 1 Medical Microbiology Research Center, Qazvin University of Medical Sciences, Qazvin, Iran; 2 Student Research Committee, Qazvin University of Medical Sciences, Qazvin, Iran; 3 Department of Pathobiology, Faculty of Medical School, Qazvin University of Medical Sciences Qazvin, Kowsar Medical and Educational Center, Qazvin, Iran

**Keywords:** High-risk HPV, HPV genotype distribution, Cervical cancer screening

## Abstract

**Background and aim:**

Persistent infections with high-risk human papillomavirus (HR-HPV) are linked to cervical cancer progression. The prevalence and distribution of HPV genotypes vary across regions and lesion severity. Comprehensive data on HPV genotype distribution among Iranian women is limited. This study investigates the distribution of HR-HPV genotypes in women with abnormal cytology in Qazvin province, northwest Iran, from 2007 to 2019.

**Materials and methods:**

A total of 103 samples, including benign cases, Low-grade Squamous Intraepithelial Lesions (LSIL), High-grade Squamous Intraepithelial Lesions (HSIL), and Invasive Cervical Cancer (ICC), were analyzed using real-time PCR to detect HPV types 16, 18, 31, 33, 35, 39, 45, 51, 52, 58, and 59.

**Results:**

The study revealed a high HPV prevalence (92.23%), with HPV-16 being the most common genotype (66.31%), followed by HPV-45 (49.47%), HPV-33 (41.05%), HPV-31(30.52%) and HPV-52 (23.15%). HPV-18 was detected only in 3 (3.15%) of cases. Of the HPV-positive samples, 82.11% had multiple infections, with HPV-16, HPV-33, and HPV-45 more prevalent in these cases. HPV-16 was significantly associated with severe lesions, particularly in ICC cases (92%, *P* = 0.007).

**Conclusion:**

These findings emphasize the role of HPV genotyping in assessing cervical lesion severity and oncogenic risk, highlighting HPV-16 as the dominant genotype across various lesion grades. The study suggests that HPV-33 and HPV-45 may also contribute significantly to cervical lesion progression.

## Introduction

Cervical cancer ranks as the fourth most common cancer among women globally and the seventh most prevalent cancer overall. In 2020, over 604,000 women were diagnosed with cervical cancer worldwide, with nearly 342,000 deaths. While developed countries have seen significant declines in cervical cancer incidence due to effective preventive measures, the disease remains a pressing health issue in developing and low-income nations due to inadequate screening and follow-up programs [1, 2]. With a rising incidence rate of 4.5 per 100,000 persons, Iran is among the countries facing this challenge [3, 4].

Persistent human papillomavirus (HPV) infection is a critical factor in the development of high-grade cervical lesions, which has driven the establishment of global cervical cancer prevention strategies [5, 6]. For decades, cervical cytology has served as the gold standard for cervical cancer screening [7]. However, WHO has updated its guidance for the screening and treatment of cervical cancer, suggesting that HPV DNA tests may be more effective than cytology, especially in low- and middle-income countries, because they are less prone to quality problems [8]. While global trends show a significant decline in cervical cancer incidence due to effective preventive measures [9, 10], Iran, like many developing countries, continues to face challenges with rising cervical cancer rates due to inadequate screening and vaccination programs [3].

Despite the existence of over 200 distinct HPV genotypes, types 16 and 18 are regarded as common high-risk HPV (HR-HPV), while genotypes 31, 33, 35, 39, 45, 51, 52, 53, 56, 58, 59, 66, 67, 68, 69, 70, 73, 82 and 85 are identified as less common HR-HPV associated with cervical cancer [11]. While HPV types 16 and 18 are predominant [12–17], other HR-HPV genotypes exhibit significant variation in their distribution, collectively contributing to approximately 30% of invasive cervical cancer cases worldwide [18].

Currently, three types of prophylactic HPV vaccines are available: a bivalent vaccine that targets HPV-16 and 18, a quadrivalent vaccine designed to protect against HPV-6, 11, 16, and 18, and a nonavalent vaccine covering HPV-6, 11, 16, 18, 31, 33, 45, 52, and 58 [19]. The prevalence and distribution of HPV genotypes vary among countries and even across regions within the same country, influenced by factors such as migration, sexual behavior, the type of cervical lesion in the studied population, and the diagnostic methods employed [20, 21].

The geographical variation in HPV prevalence and infection patterns has significant implications for tailoring region-specific HPV DNA testing, vaccine selection, and assessing expected efficacy. Countries that have implemented widespread prophylactic HPV vaccination and comprehensive cervical cancer screening programs have achieved substantial reductions in cervical cancer incidence and mortality [22]. This study presents comprehensive findings on HR-HPV genotyping in women with abnormal cytology from Qazvin province, Iran.

## Materials and methods

### Patients and sampling

A total of 111 formalin-fixed, paraffin-embedded (FFPE) cervical biopsy blocks were retrospectively collected from the pathology archives of Qazvin Kowsar Referral Gynecology Hospital, spanning the years 2007 to 2019. Each sample had a confirmed diagnosis through cytological or histological evaluation. Diagnoses included Invasive Cervical Cancer (ICC), characterized as either Squamous Cell Carcinoma (SCC) or Adenocarcinoma (AD). Additionally, samples represented various stages of Cervical Intraepithelial Neoplasia (CIN), ranging from grade 1 to grade 3, as well as LSIL and HSIL.

### DNA extraction

For DNA extraction, a five-micron section was cut from each FFPE cervical biopsy block using a microtome (Hacker instrument, USA). DNA extraction was carried out with the High Pure FFPE Micro DNA Kit (Roche Diagnostics, Indianapolis, IN, USA). The quality of the extracted DNA was assessed by Nanodrop spectrophotometer and β-globin gene detection. For β-globin detection, PCR was performed on an Applied Biosystems Veriti 96-well Fast Thermal Cycler (CA, USA) using PC04 and GH20 primers [23]. Briefly, 5 µl of genomic DNA was mixed with 12.5 µl Red Taq Master Mix (Genaxxon bioscience), 0.5 µl of each primer, and 6.5 µl PCR-grade water. The PCR conditions were as follows: Initial denaturation at 94 °C for 10 min, denaturation at 94 °C for 1 min, annealing at 40 °C for 1 min, and extension at 72 °C for 90 s.

### HPV detection

Samples positive for β-globin were screened for the presence of the HPV genome using Real-Time PCR on the StepOnePlus Real-Time System (Applied Biosystems, CA, USA) with GP5+/6 + primers [24]. Each amplification reaction was performed in a total volume of 20 µl, comprising 0.5 µl of each primer, 10 µl TAKARA Sybr Premix Ex Taq (Takara, Otsu, Japan), 0.4 µl ROX Reference Dye, 6.6 µl PCR-grade water, and 2 µl of extracted DNA.

The PCR cycling conditions were as follows: initial denaturation at 95 °C for 2 min, denaturation at 94 °C for 30 s, annealing at 40 °C for 2 min, and extension at 72 °C for 90 s.

### Genotyping

Real-time PCR using type-specific primers was performed for the detection of HPV types 16, 18, 31, 33, 45, 52 and 58 (Table 1). The primers were designed using the Integrated DNA Technologies platform (IDT, Iowa, USA). Each reaction, with a final volume of 20 µl, included 0.5 µl of each primer, 10 µl TAKARA Sybr Premix Ex Taq, 0.4 µl ROX Reference Dye, 6.6 µl PCR-grade water, and 2 µl of extracted DNA. Thermal cycling conditions were as follows: 60 s at 95 °C, followed by 30 s at 95 °C, 30 s at 60 °C, and 30 s at 72 °C. Additionally, all PCR products were subjected to gel electrophoresis to validate the PCR reactions through band size analysis.

**Table 1:** Primer sequences for HPV genotyping

HPV type	Primer and probe sequences (5’ to 3’)
16	F: TGCTAGTGCTTATGCAGCA R: TTACTGCAACATTGGTACATGG
18	F: CGCCACGTCTAATGTTTCTG R: CCTGTGATAAAGGACGCGA
31	F: CTCTAATAGATATGCCGGTGGTC R: CCCAATGCTCTCCAATAGGT
33	F: ACAAGTATCCTGGACAACCG R: CAGGTGCTGCATTAGTACAAG
35	F: CAAGAATTACAGCGGAGTGAGGTA R: ATATGGCTGGCCTTCTCTATATACTATACA P: VIC- ATGACTTTGCATGCTATG -MGB
39	F: AGTCTGCAGCCATTACATGTCAA R: GTCAACCGGCTGTATTTCATTGT P: FAM- AGGATGCTCCAGCACC -MGB
45	F: AGTGCTCATGCAGCTACAG R: CCAGGTTGCAATTGTGCAG
51	F: TGCCACTGCTGCGGTTT R: CCCCATGCCTAATATATTGCTTAAA P: FAM- CCCAACATTTACTCCAAG -MGB
56	F: ATGCATGGTAAAGTACCAACGCT R: TGTAGGTCAATTTCTGTTTGAGGTG P: VIC- CAAGACGTTGTATTAGAACTA -MGB
52	F: GCCGGTACCTTAGGTGACC R: GCTTTGTACAGTGGCAGTATTG
58	F: TCCAGGACGCAGAGGAGAA R: TCCAACGCCTGACACAAATC P: NED- CCACGGACATTGCA -MGB
59	F: AGCGACCAAGACAGTGTGGAT R: GTCCACTGACACGCTGGTAGAC P: NED- CACACAGCACCCTC -MGB

Multiplex Real-Time PCR using type-specific primers and probes was conducted to detect additional HR-HPV types, including types 35, 39, 51, 56, and 59 (Table 1) [25]. For this purpose, three separate reaction tubes were prepared: tube 1 for types 56 and 58, tube 2 for types 39 and 35, and tube 3 for types 51 and 59.

Each reaction mixture had a final volume of 20 µl, consisting of 4 µl DNA, 8 µl amplicon, and 4 µl primer/probe mixture. The primer/probe mixture included 500 nM of each primer and 100 nM of each probe. PCR reactions were performed on the Qiagen Rotor-Gene system with the following thermal cycling conditions: initial denaturation at 95 °C for 15 min, followed by denaturation at 95 °C for 10 s, annealing at 60 °C for 15 s, and extension at 72 °C for 15 s.

### Statistical analysis

The collected data were summarized and presented in statistical tables and as numerical indices. Statistical analysis was performed using SPSS for Windows, version 21.0 (IBM, USA). The Chi-square or Fisher exact tests were applied to analyze categorical data. A P-value less than 0.05 was considered statistically significant.

## Results

### Sample analysis

A total of 103 samples, including 51 benign, 17 LSIL, 10 HSIL, and 25 ICC samples, were examined further after excluding eight samples due to unacceptable NanoDrop results. All remaining samples tested positive for β-globin DNA.

### HPV detection and genotyping

Among these, 95 (92.23%) samples were positive for HPV DNA. The most prevalent genotype was HPV-16 (*n* = 63, 66.31%), followed by HPV-45 (*n* = 47, 49.47%) and HPV-33 (*n* = 39, 41.05%). Other frequently detected genotypes included HPV-31 (*n* = 30, 31.57%) and HPV-52 (*n* = 22, 23.15%). Less common genotypes were HPV-18 (*n* = 3, 3.15%), HPV-58 (*n* = 2, 2.1%), and HPV-39 (*n* = 1, 1.05%). HPV types 35, 51, and 59 were not detected in any sample (Table 2).

**Table 2:** Prevalence of high-risk HPV genotypes among HPV-positive samples

HPV Genotype	Number of Cases (*n*)	Prevalence (%)
HPV-16	63	66.31%
HPV-45	47	49.47%
HPV-33	39	41.05%
HPV-31	30	31.57%
HPV-52	22	23.15%
HPV-18	3	3.15%
HPV-58	2	2.10%
HPV-39	1	1.05%
HPV-35, 51, 59	0	0%

### Single vs. multiple infections

Among the 95 HPV-positive samples (Table 3), only 17 (17.89%) had a single HPV infection, while the majority (78 samples, 82.11%) had multiple HPV infections. HPV-16 was the most frequent genotype in both single and multiple infections, with a higher frequency in multiple infections (72.97%). Additionally, HPV-33 and HPV-45 were significantly more frequent in multiple infections compared to single infections (*P* = 0.032 and *P* = 0.031, respectively, using the Chi-square test), suggesting that these genotypes may be associated with a higher infection burden. Conversely, HPV-39 and HPV-58 were detected exclusively in single infections, although their frequency was low.

**Table 3:** Prevalence of HPV types in single and multiple infections

HPV type	Single infection	Multiple infections	*P* value
16	9 (52.94%)	54 (72.97%)	0.747
18	0	3 (4.05%)	1.000
31	0	29 (39.18%)	0.003
33	2 (11.76%)	37 (50%)	0.032
39	1 (5.88%)	0	1.000
45	3 (17.64%)	44 (59.45%)	0.031
52	0	22 (29.72%)	0.032
58	2 (11.76%)	0	1.000
Total	17 (17.89%)	78 (82.11%)	0.0001

### Analysis of HPV genotype distribution

When comparing the prevalence of HPV genotypes across different pathological grades (Table 4), significant associations were observed for HPV-16 and HPV-33. The prevalence of HPV-16 increased significantly with the severity of the lesion, being most frequent in ICC cases (92%, *P* = 0.007). On the other hand, HPV-33 was most commonly detected in benign cases (53.48%, *P* = 0.007). Although HPV-18 was detected in 12% of ICC cases, its overall prevalence remained low, reflecting the regional differences in genotype distribution. The patients’ ages ranged from 20 to 75 years. There were not any significant differences between the prevalence of HPV genotypes in different age groups. The prevalence of HPV genotypes in different age groups is depicted in Table 5.

**Table 4:** Prevalence of HPV types in different pathological grades

HPV type	Benign *N* = 51	LSIL/CIN1 *N* = 17	HSIL/CIN2+ *N* = 10	ICC *N* = 25	*P* value
16	22 (51.16%**)**	11 (64.70%**)**	7 (70%**)**	23 (92%**)**	0.007
18	0	0	0	3 (12%)	1.000
31	12 (27.90%)	5 (29.41%)	2 (20%)	11 (44%)	0.338
33	23 (53.48%)	7 (41.17%)	5 (50%)	3 (12%)	0.007
39	0	0	1 (10%)	0	1.000
45	22 (51.16%)	10 (58.82%)	3 (30%)	11 (44%)	0.489
52	13 (30.23%)	5 (29.41%)	2 (20%)	1 (4%)	0.073
58	0	1 (5.88%)	0	0	1.000
HPV+	43 (84.31%)	17 (100%)	10 (100%)	25 (100%)	0.360

**Table 5:** Prevalence of HPV genotypes in different age groups

HPV type	Age group						*P* value
	20–29 *n* = 13	30–39 *n* = 24	40–49 *n* = 32	50–59 *n* = 13	60–69 *n* = 8	70–79 *n* = 2	
16	9 (69.2%)	10 (52.6%)	17 (56.6%)	10 (83.3%)	8 (100%)	2 (100%)	0.059
18	0	0	0	1 (8.3%)	1 (12.5%)	1 (50%)	1.000
31	4 (30.7%)	6 (31.5%)	12 (40%)	2 (16.6%)	4 (50%)	0	0.425
33	7 (53.8%)	5 (26.3%)	14 (46.6%)	4 (33.3%)	0	0	0.162
39	0	0	1 (3.1%)	0	0	0	1.000
45	8 (61.5%)	10 (52.6%)	17 (56.6%)	3 (25%)	5 (62.5%)	1 (50%)	0.232
52	4 (30.7%)	3 (15.7%)	9 (30%)	0	0	0	1.000
58	0	0	1 (3.1%)	0	0	0	1.000
HPV+	13 (100%)	19 (79.1%)	32 (100%)	12 (92.3%)	8 (100%)	2 (100%)	0.340

## Discussion

Cervical cancer is still the fourth most frequent cause of death among women globally [26]. A prolonged infection with HPV strains is the primary cause of nearly all cases of cervical cancer [27]. Examining the prevalence of HPV genotypes in women from specific regions and their correlation with cervical lesions can help improve cervical cancer screening programs and guide HPV vaccination strategies [28]. Hence, this study aims to assess the distribution of HPV genotypes among women with abnormal cytology results in Qazvin province, located in northwest Iran.

In this study, 92.23% of the samples tested positive for HPV DNA, with HPV-16 being the most prevalent genotype, followed by HPV-45 and HPV-33. These findings are in line with previous studies emphasizing HPV-16 as the dominant genotype in cervical carcinogenesis [29–31]. The other most common HPV genotypes in cervical cancers among Qazvinian women were HPV-33 and HPV-45, which differ from the frequently occurring HR-HPV genotypes in other countries [32].

Among the 95 HPV-positive samples, multiple infections were more common (82.11%) than single infections (17.89%). HPV-16 was the most prevalent genotype in both groups, with a higher frequency in multiple infections. Additionally, HPV-33 and HPV-45 were significantly associated with multiple infections, while HPV-39 and HPV-58 were found only in single infections. These findings indicate that certain genotypes may be more likely to cause multiple infections and could play a significant role in the overall infection burden. This observation aligns with studies that have reported a high incidence of multiple HPV infections in similar cohorts [33–35]. Multiple infections have also been shown to be associated with a higher viral load and a greater likelihood of developing severe cervical lesions [36]. This suggests that multiple infections may increase the risk of progression to cervical cancer [37]. The clinical implications of these findings are important for both screening and treatment strategies [38]. In areas where multiple infections are common, it may be beneficial to implement more sensitive and specific HPV genotyping techniques to identify co-infections more effectively [39]. Moreover, treatment strategies may need to be adapted to address the increased infection burden associated with multiple HPV types [40].

HPV-16 prevalence significantly increased with lesion severity, reaching 92% in ICC cases (*P* = 0.007). HPV-33 also showed a significant association across pathological grades, aligning with global findings [41, 42].

HPV-33 was most prevalent in benign cases (53.48%, *P* = 0.007), while HPV-18, though present in 12% of ICC cases, had a low overall prevalence, highlighting regional genotype differences. This contrasts with studies from Tehran and Isfahan, where HPV-18 was more frequently linked to cervical cancers [29, 43]. The relatively low prevalence of HPV-18 (3.15%) in our study warrants further investigation, as it is typically seen as the second most common genotype in cervical cancers [41, 44].

The high frequency of multiple infections (82.11%) observed in this study is noteworthy. This is considerably higher than what was reported by Tabibzadeh et al. (43.36%) and Salehi-Vaziri et al. (33.84%) [33, 34]. Multiple infections have also been observed in other regions, such as Isfahan, where 83.1% of HPV-positive samples exhibited multiple infections [29]. We believe this emphasizes the importance of employing advanced genotyping techniques to better understand the clinical implications of multiple HPV infections, particularly in socio-cultural contexts where most women may have a single sexual partner.

Our analysis of different cytological categories revealed that HPV prevalence in HSIL samples (100%) exceeded reported rates in Iran (69%), Asia (81%), and worldwide (84.2%) [41, 42, 44]. HPV-16 was the most frequently detected genotype in HSIL and ICC samples, consistent with global meta-analyses [41, 42]. However, the high prevalence of HPV-33 in benign lesions observed in this study suggests that HPV-33 may play an early role in the neoplastic progression of cervical lesions, which warrants further exploration [33].

Geographical variations in genotype prevalence were also noted in LSIL samples. While our study identified HPV-16, 45, and 33 as the most prevalent genotypes in this category, meta-analyses by Salvatiha et al. and Bao et al. [41, 44] have reported HPV-16, 18, and 39 as the most common types in Iranian and Asian populations, respectively. These discrepancies emphasize the need for region-specific HPV surveillance to optimize preventive measures, such as vaccination, especially in areas with varying genotype distributions.

In this study, we employed two different real-time PCR approaches—SYBR Green-based and probe-based multiplex systems—for HPV genotyping. The SYBR Green method was used to detect common HR-HPV types such as HPV-16, 18, 31, 33, 45, 52, and 58 due to its simplicity and cost-effectiveness. To identify less frequently reported types including HPV-35, 39, 51, 56, and 59, we used a multiplex probe-based PCR, which offers greater specificity and the ability to detect multiple genotypes simultaneously. While differences in methodology may affect sensitivity and specificity, rigorous quality control and the use of type-specific primers helped minimize inconsistencies. This dual-method approach enabled comprehensive and cost-efficient genotyping, which is especially relevant in resource-limited settings like ours.

Lastly, the age distribution of HPV genotypes did not show significant differences, although the predominance of HPV-16 across all age groups aligns with its established oncogenic potential. This reinforces the critical need for early HPV screening, particularly as HPV-16 has a higher tendency to persist and induce malignancy than other genotypes [33].

In conclusion, the predominance of HPV-16, 45, and 33 among women with abnormal cytology in Qazvin province underscores the need for localized vaccination strategies. The nonavalent HPV vaccine, which covers HPV-16, 33, and 45, may offer optimal protection for this population. However, the low prevalence of HPV-18 and the high rate of multiple infections highlight the importance of region-specific epidemiological studies to tailor preventive measures effectively. Further research is essential to assess the long-term impact of these findings on cervical cancer prevention and control.

### Limitations and future directions

This study’s limitations include its relatively small sample size and the retrospective nature of data collection, which may limit the generalizability of findings to other regions. Additionally, the study was confined to a single province, potentially overlooking broader genotype distribution patterns in Iran. Future research should incorporate larger, multi-center studies to address these gaps and further elucidate HPV epidemiology across different regions.

## Data Availability

No datasets were generated or analysed during the current study.
